# Overworking

**Published:** 1919-05

**Authors:** 


### Overworking.

Every once in while some one gets into print with the statement that people cannot overwork—that work is good for every one and that worry is the only thing that kills prematurely. Such statements are likely to do incalculable harm. This has been proven by the great number of useful physicians who have killed themselves or permanently impaired their health by working night and day through the influenza epidemic without sufficient rest. That epidemic, which was one of the worst in the history of the country, came when a large number of the doctors were in the service of the country, and an unusual burden was thrown on those who had to handle it. They responded to every call possible with all the heroism shown by veterans of the battlefields, and many of them virtually sacrificed their lives in doing so, taxing their strength beyond the limit of physical endurance. Others have been so exhausted through their constant attention to their patients that they will be months in recovering their normal strength, if they ever do.

These men, many of them, are in need of the vacations that they are recommending to their patients. They owe it to themselves and to those whom they are serving to take the best of care of themselves. They can't work all the while under the severe strain under which they are going and remain efficient. There was once a Yankee apiarist who conceived the idea of making his bees work all the year. He would keep them in Northern Indiana in the summer and in Florida in the winter and have them busy all the while. The result was that the bees soon worked themselves to death. Some doctors who would not permit a patient to overwork if they could help it are going without the rest they need so much and now and then one falls by the wayside prematurely.

Many of the young doctors are returning from the service. It is true that they have been under a long strain, but they are anxious to get back into the practice, which to them will be a rest. Why not turn over your practice, doctor, to one of these men and go somewhere for a long summer rest? It will do both of you good and you will be putting into practice some of your own advice.

